# Omega-3 Fatty Acid Supplementation Improves Endothelial Function in Primary Antiphospholipid Syndrome: A Small-Scale Randomized Double-Blind Placebo-Controlled Trial

**DOI:** 10.3389/fimmu.2018.00336

**Published:** 2018-03-02

**Authors:** Sheylla M. Felau, Lucas P. Sales, Marina Y. Solis, Ana Paula Hayashi, Hamilton Roschel, Ana Lúcia Sá-Pinto, Danieli Castro Oliveira De Andrade, Keyla Y. Katayama, Maria Claudia Irigoyen, Fernanda Consolim-Colombo, Eloisa Bonfa, Bruno Gualano, Fabiana B. Benatti

**Affiliations:** ^1^Applied Physiology and Nutrition Research Group, Laboratory of Assessment and Conditioning in Rheumatology, Faculdade de Medicina FMUSP, Universidade de Sao Paulo, Sao Paulo, Brazil; ^2^Heart Institute (InCor), Faculdade de Medicina FMUSP, Universidade de Sao Paulo, Sao Paulo, Brazil; ^3^School of Applied Sciences, Universidade Estadual de Campinas (UNICAMP), Limeira, Brazil

**Keywords:** antiphospholipid syndrome, n-3 PUFA, endothelial function, coagulation, inflammation

## Abstract

Endothelial cells are thought to play a central role in the pathogenesis of antiphospholipid syndrome (APS). Omega-3 polyunsaturated fatty acid (n-3 PUFA) supplementation has been shown to improve endothelial function in a number of diseases; thus, it could be of high clinical relevance in APS. The aim of this study was to evaluate the efficacy of n-3 PUFA supplementation on endothelial function (primary outcome) of patients with primary APS (PAPS). A 16-week randomized clinical trial was conducted with 22 adult women with PAPS. Patients were randomly assigned (1:1) to receive placebo (PL, *n* = 11) or n-3 PUFA (ω-3, *n* = 11) supplementation. Before (pre) and after (post) 16 weeks of the intervention, patients were assessed for endothelial function (peripheral artery tonometry) (primary outcome). Patients were also assessed for systemic markers of endothelial cell activation, inflammatory markers, dietary intake, international normalized ratio (INR), and adverse effects. At post, ω-3 group presented significant increases in endothelial function estimates reactive hyperemia index (RHI) and logarithmic transformation of RHI (LnRHI) when compared with PL (+13 vs. −12%, *p* = 0.06, ES = 0.9; and +23 vs. −22%, *p* = 0.02, ES = 1.0). No changes were observed for e-selectin, vascular adhesion molecule-1, and fibrinogen levels (*p* > 0.05). In addition, ω-3 group showed decreased circulating levels of interleukin-10 (−4 vs. +45%, *p* = 0.04, ES = −0.9) and tumor necrosis factor (−13 vs. +0.3%, *p* = 0.04, ES = −0.95) and a tendency toward a lower intercellular adhesion molecule-1 response (+3 vs. +48%, *p* = 0.1, ES = −0.7) at post when compared with PL. No changes in dietary intake, INR, or self-reported adverse effects were observed. In conclusion, 16 weeks of n-3 PUFA supplementation improved endothelial function in patients with well-controlled PAPS. These results support a role of n-3 PUFA supplementation as an adjuvant therapy in APS. Registered at http://ClinicalTrials.gov as NCT01956188.

## Introduction

Antiphospholipid syndrome (APS) is a systemic autoimmune disease characterized by recurrent thrombotic episodes and/or obstetric morbidities and persistent serum antiphospholipid antibodies (aPL). APS can be classified as primary or secondary if concurrent with another autoimmune disease, tumor, or hematologic disorder (1). Despite adequate anticoagulant treatment, primary APS (PAPS) is significantly associated with high morbidity and mortality from vascular thrombotic events (2) and an increased risk of cardiovascular diseases (CVDs) (3).

Previous studies propose that endothelial cells play a central role in the pathogenesis of APS (4). aPLs have been shown to bind to endothelial cell beta-2 glycoprotein I (β2GPI) receptors leading to endothelial malfunction and formation of thrombosis (5). Evidence exists that patients with APS show an impaired endothelial function when compared with their healthy peers (6, 7). Although not fully elucidated, potential underlying mechanisms include aPL-mediated endothelial cell activation due to increases in the production and release of adhesion-cell molecules and pro-inflammatory cytokines (4, 8), particularly tumor necrosis factor (TNF) and interleukin (IL)-1β (8, 9). Endothelial dysfunction is the earliest detectable stage predisposing to the formation of atherosclerotic lesions and cardiovascular events (10). Thus, strategies capable of minimizing endothelial dysfunction may be of high clinical relevance in APS.

Supplementation of marine-derived omega-3 polyunsaturated fatty acids (n-3 PUFA), eicosapentaenoic acid (EPA), and docosahexanoic acid (DHA) (≥2.0 g/day) has been shown to have antiatherogenic and antithrombotic properties *via* improvements in endothelial function in type 2 diabetes mellitus (T2D) and dyslipidemia (11–14), which are conditions associated with accelerated atherosclerosis and an increased CVD risk (15, 16). Supplementation of marine n-3 PUFA may also be beneficial in autoimmune rheumatic diseases, since the intake of 3 g/day of EPA and DHA has been shown to improve clinical features, disease activity, and endothelial function in systemic lupus erythematosus (SLE) patients (17). However, no studies have assessed the potential beneficial effects of n-3 PUFA supplementation in APS.

The aim of the present study was to evaluate the efficacy of n-3 PUFA supplementation on endothelial function (primary outcome) in patients with PAPS. Secondary outcomes were systemic inflammation and lipid profile.

## Methods

### Experimental Design

A 16-week randomized clinical trial was conducted between May 2014 and November 2016 in São Paulo, SP, Brazil (registered at http://ClinicalTrials.gov as NCT01956188). This manuscript is reported according to the CONSORT guidelines and approved by the Ethics Committee for Analysis of Research Projects of the General Hospital, School of Medicine, University of São Paulo, affiliated to the National Committee for Ethics in Research of Brazil. Patients were randomly assigned (1:1) to receive either placebo (PL, *n* = 11) or n-3 PUFA (ω-3, *n* = 11) supplementation according to a computer-generated treatment sequence in a double-blind design. Before (pre) and after (post) 16 weeks of intervention, patients were assessed for endothelial function using peripheral artery tonometry (primary outcome). Patients were also assessed for systemic markers of endothelial cell activation [intercellular adhesion molecule-1 (ICAM-1), vascular adhesion molecule-1 (VCAM-1), e-selectin, and fibrinogen], systemic inflammatory markers [C-reactive protein (CRP), IL-6, IL-10, TNF, IL-1ra, and IL-1β], lipid profile, dietary intake, international normalized ratio (INR), and self-reported adverse effects. At pre, patients were also assessed for physical activity level (using the short version of the International Physical Activity Questionnaire) for characterization purposes (18). Blood collection and endothelial function assessments were performed on the same day. Patients were instructed to maintain habitual physical activity and food intake throughout the study.

### Patients

Patients were recruited from the outpatient APS clinic of the Rheumatology Division, School of Medicine, University of São Paulo. Sample consisted of 22 adult women (aged 27–45 years) diagnosed with PAPS according to the international criteria (1). Exclusion criteria were as follows: age >45 years; body mass index (BMI) ≥35 kg/m^2^; secondary APS; menopause or amenorrhea; pregnancy or lactation; prednisone current use or in the 3 months before entering the study; previous n-3 PUFA supplementation; chronic use of anti-inflammatory drugs; hemorrhagic or thrombotic episode in the 6 months before entering the study; untreated thyroid dysfunction; uncontrolled hypertension; T2DM; treatment with statins, fibrate, insulin, or insulin sensitizers; tobacco use; acute renal failure, hepatic, cardiac, and pulmonary involvement. This study was approved by the Ethics Committee for Analysis of Research Projects of the General Hospital, School of Medicine, University of São Paulo, affiliated to the National Committee for Ethics in Research of Brazil. All subjects signed the informed consent prior to participation.

### Supplementation Protocol and Blinding Procedure

The ω-3 group received 1.8 g of EPA and 1.3 g of DHA as re-esterified triglycerides contained in five capsules (three capsules of HiOmega-3 + two capsules of Omega-3 DHA 500, Naturalis^®^, São Paulo, SP, Brazil) which were consumed once per day. EPA and DHA contained in the capsules were extracted from whole fish oil. The same dose and proportion of EPA and DHA has been shown to beneficially impact endothelial function in patients with T2D (11), chronic heart failure (19), and SLE patients (17). PL group received the exact same amount of capsules per day, similar in size, shape, and color, containing soy oil. Supplement packages were coded so that neither patients nor investigators were aware of the content until completion of analyses. Compliance to supplementation was monitored weekly.

### Endothelial Function Estimates

Endothelial function was assessed *via* peripheral artery tonometry using the EndoPAT-2000 device (Caesarea, Israel). This is a reactive hyperemia peripheral arterial plethysmography which measures the vascular endothelial response to temporary vascular deprivation in the arm by measuring blood volume in the finger. To that end, a probe was applied to a finger in each hand, whereas an inflatable blood pressure cuff was applied to one upper arm. A baseline measurement of finger volumes was recorded for 5 min. Then, the arm cuff was inflated to either 60 mm Hg above systemic systolic blood pressure or 200 mm Hg (whichever was higher) for 5 min. When the cuff was deflated, changes in finger blood volume were recorded from each finger for another 4 min. In healthy subjects, the period of circulatory deprivation in an arm is followed by a marked vasodilatation in the ipsilateral finger 90–120 s after cuff deflation which is due to a local release of NO and prostaglandins as a response to shear stress and/or tissue hypoxia. Differences between the pre- and post-occlusion finger blood volume in each hand are used to calculate the reactive hyperemia index (RHI), which is a measure of the endothelial response to occlusion and reactive hyperemia corrected for systemic effects on the basis of volume changes in the contralateral finger. The augmentation index (AI), an estimate of vascular stiffness, is also automatically calculated, as well as the logarithmic transformation of RHI (LnRHI). Patients were instructed to refrain from physical exercise, alcohol, and caffeine intake 24 h prior to the test. Moreover, they consumed a standardized meal 1 h prior to testing.

### Blood Analysis

Blood samples were collected after a 10-h overnight fast. Endothelial function markers (ICAM-1, VCAM-1, and e-selectin) and inflammatory cytokines (IL-10, IL-6, IL-1-β, IL-1-ra, and TNF) were measured *via* immunoassays using multiplex human panels according to the manufacturer’s procedures (Milliplex^®^, USA). Serum levels of fibrinogen were determined using the Clauss method, whereas serum levels of CRP were measured using the immunoturbidimetry method (Cobas 8000). Plasma levels of blood cholesterol, high-density lipoprotein (HDL)-cholesterol, and triglycerides were assessed *via* colorimetric enzymatic methods (CELM, Brazil). From these, very-low lipoprotein-cholesterol (VLDL-cholesterol) (VLDL-cholesterol = triglycerides/5) and low-density lipoprotein (LDL) cholesterol [LDL-cholesterol = total cholesterol − (HDL-cholesterol + VLDL-cholesterol)] levels were calculated. Prothrombin time was measured in an automated coagulometer (3000 IL) with the use of bovine thromboplastin and was expressed as INR.

### Dietary Intake

Dietary intake was assessed using three 24-h dietary recalls undertaken on separate days (2 weekdays and 1 weekend day) using a visual aid photo album of real foods. Energy and macronutrient intake were analyzed by Avanutri software (Rio de Janeiro, Brazil). n-3 PUFA intake was estimated based on the content of DHA, EPA, and α-linoleic acid (ALA) of foods according to the United States Department of Agriculture National Nutrient Database for Standard Reference available at https://ndb.nal.usda.gov/ndb/search/list.

### Statistical Analysis

To minimize the impact of inter-individual variability, all values were converted into delta scores (i.e., post–pre-values) and thereafter tested by a mixed model, having pre-values from all dependent variables as covariates. Tukey *post hoc* was used for multiple comparisons. Baseline data were compared using Fisher’s exact tests and unpaired Student’s *t*-tests. Fisher’s exact tests were also used to compare adherence to supplementation, whereas McNemar’s test and Fisher’s exact tests were used to compare within- and between-group proportion changes in lipid profile. Cohen’s *d* was used to determine between-group effect sizes (ES) for dependent variables (20). Data are presented as mean (standard deviation), difference between delta changes, and 95% confidence interval (95% CI) unless otherwise stated. The significance level was set at *p* ≤ 0.05, with a trend toward significance being accepted at *p* ≤ 0.1.

*Post hoc* power analyses were performed with the assistance of the G-Power^®^ software (version 3.1.2), which demonstrated a power of 65 and 73% at an alpha level of 5% to detect significant differences in RHI and LnRHI between PL and ω-3 with ES of 0.9 and 1.0.

## Results

### Patients and Adherence to the Supplementation Protocol

**Figure 1:**
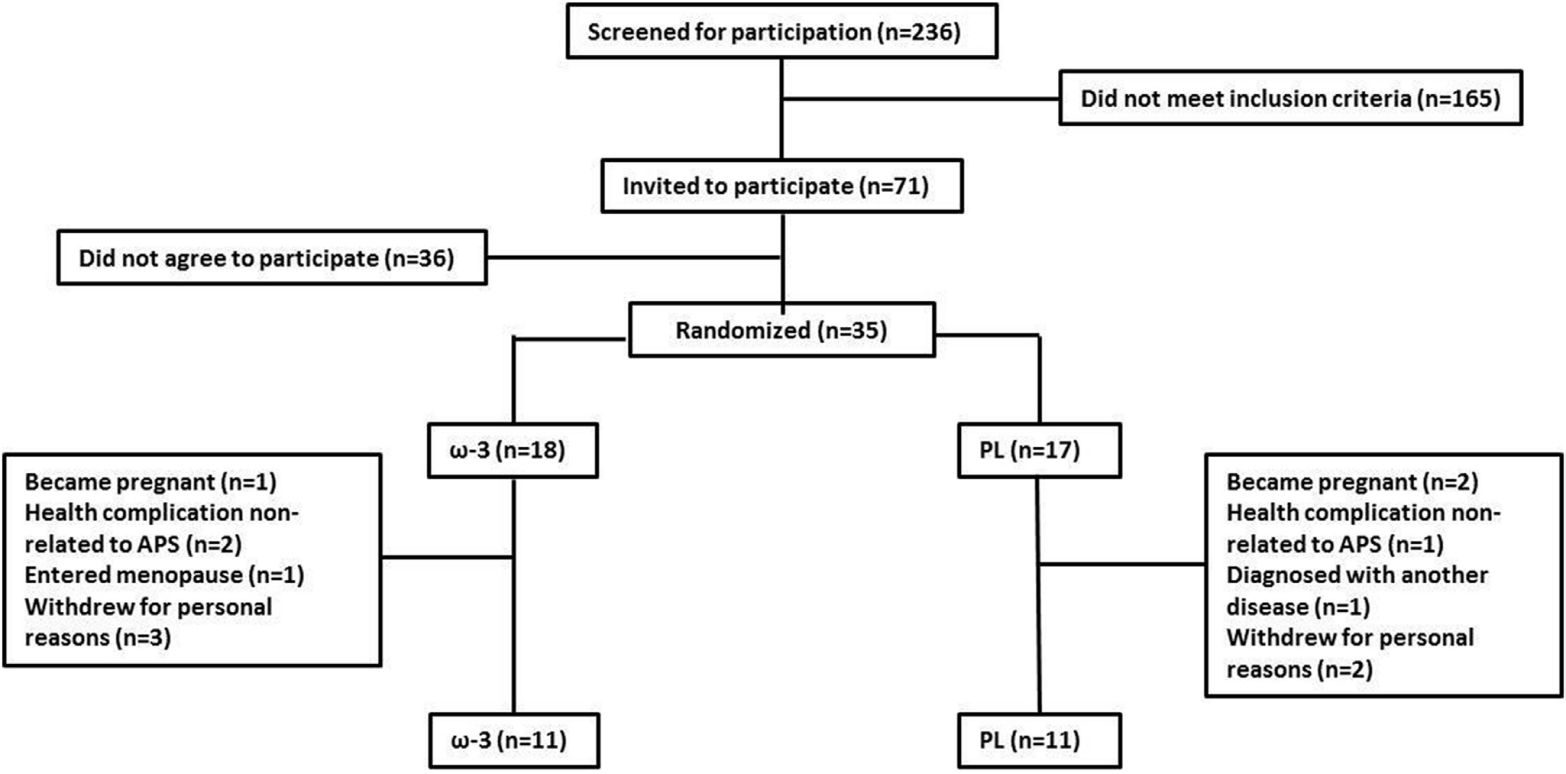
Flow diagram of patients. Symbol abbreviation: APS, antiphospholipid syndrome.

Two-hundred and thirty-six patients were screened for participation and 71 met the inclusion criteria. Thirty-five agreed to take part in the study and were randomly assigned to either the ω-3 (*n* = 18) or PL (*n* = 17) group. Five patients withdrew from the study for personal reasons, three patients became pregnant, one patient entered menopause, one patient was diagnosed with another autoimmune disease, and three patients experienced disease complications not related to APS. Thus, the 22 patients who completed the study were analyzed (ω-3 = 11, PL = 11) (Figure 1). We chose a “per protocol” approach instead of an intention-to-treat (ITT) protocol as the primary research goal of our study was to determine the potential efficacy of n-3 PUFA supplementation and not its effectiveness (21). In this context, ITT analysis has been regarded as more susceptible to type II error (22, 23), as the treatment effect may be diluted due to dropouts (23). Importantly, baseline comparisons using Fisher’s exact tests and unpaired *t*-tests analyses of those lost to follow-up and those retained in each group did not show any dropout bias (data not shown).

Due to technical issues, two patients (one from each group) were not assessed for endothelial function. Table 1 shows the demographic characteristics at baseline. No between-group differences were observed at baseline for any of the parameters. Adherence to supplementation protocol was 83% in ω-3 and 88% in PL (*p* = 0.2, between-group comparison).

**Table 1:** Demographic and current clinical and treatment data in ω-3 and PL.

	PL (*n* = 11)	ω-3 (*n* = 11)	*p*
Age (years)	37.6 ± 6.5	34.8 ± 4.5	0.29
BMI (kg/m^2^)	28.8 ± 4.3	29.8 ± 4.3	0.55
Disease duration (years)	8.4 ± 5.8	10.0 ± 5.1	0.52
Previous thrombotic event [no. (%)]	8 (72.7)	10 (90.9)	0.59
Previous obstetric morbidity [no. (%)]	6 (54.5)	7 (63.6)	1.0
Anti-cardiolipin antibody positivity [no. (%)]	9 (81.8)	5 (45.4)	0.18
Lupic anticoagulant antibody positivity [no. (%)]	7 (63.6)	11 (100)	0.1
Anti-beta-2 glycoprotein I antibody positivity [no. (%)]	3 (27.3)	2 (18.2)	1.0
Triple antiphospholipid antibody positivity [no. (%)]	2 (18.2)	1 (9.1)	1.0
Arterial hypertension [no. (%)]	1 (9.1)	3 (27.3)	0.59

**Drug intake**			
Oral contraceptives (progestogen) [no. (%)]	5 (45.4)	4 (36.4)	1.0
Glucocorticoid [no. (%)]	0 (0)	0 (0)	1.0
Hydroxychloroquine [no. (%)]	4 (36.4)	5 (54.5)	1.0
Acetylsalicylic acid [no. (%)]	2 (18.2)	2 (18.2)	1.0
Anticoagulant drugs [no. (%)]	9 (81.8)	9 (81.8)	1.0

**Physical activity level**			
Low [no. (%)]	2 (18.2)	4 (36.4)	0.63
Moderate [no. (%)]	4 (36.4)	4 (36.4)	1.0
High [no. (%)]	5 (45.4)	3 (27.3)	0.65

*Data expressed as mean ± SD or number of patients (percentage) and level of significance (*p*) calculated using unpaired Student’s *t*-test or Fisher exact test*.

*BMI, body mass index*.

### Endothelial Function

**Figure 2:**
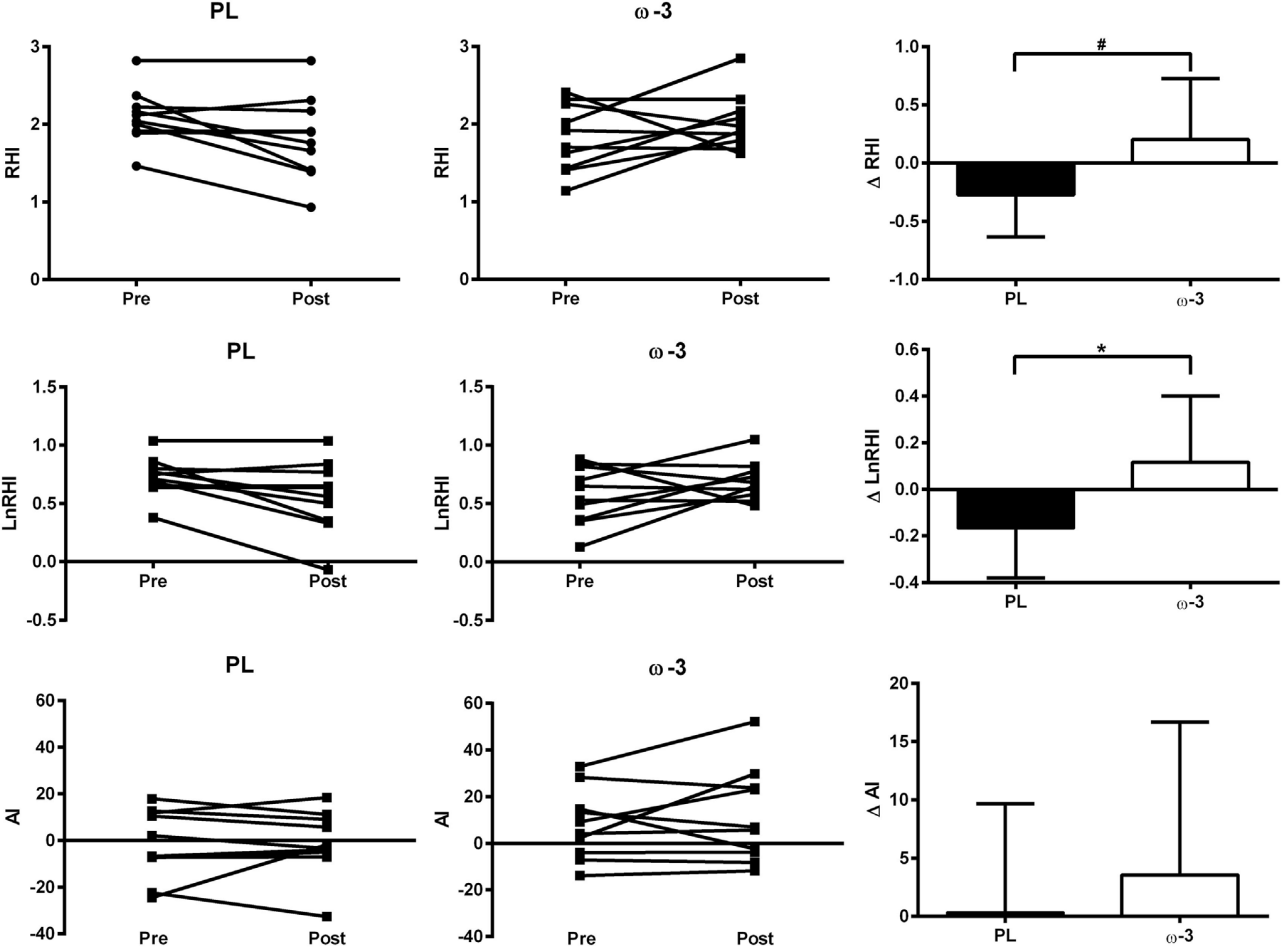
Individual data at pre and post and absolute change (data expressed as mean and standard deviation) in endothelial function estimates RHI, LnRHI, and AI in ω-3 and PL. Differences between delta changes were calculated using a mixed model adjusted by pre-values. *means *p* < 0.05, ω-3 vs. PL; ^#^means *p* = 0.06, ω-3 vs. PL. Symbol abbreviation: RHI, reactive hyperemia index; LnRHI, reactive hyperemia index after natural log transformation; AI, augmentation index.

Following the intervention, ω-3 presented significant increases in LnRHI (+23 vs. −22%, *p* = 0.02, ES = 1.0) and a tendency toward increases in RHI (+13 vs. −12%, *p* = 0.06, ES = 0.9) when compared with PL. Previous studies have shown between- and within-day coefficient variation (CV) ranging from 11 to 22% in PAT-derived measures of endothelial function (24–26). Thus, it is noteworthy that out of the 10 patients in ω-3, 6 patients showed increases and 2 patients showed decreases in endothelial function estimates above the previously reported CVs (> + 30% in RHI and > + 50% in LnRHI), whereas two patients showed no change. By contrast, out of the 10 patients in PL, 5 patients showed decreases in endothelial function estimates above the previously reported CVs (>25% in RHI and >30% in LnRHI), whereas 5 showed no change (Table 2; Figure 2). By contrast, no significant differences between ω-3 and PL were observed in AI (+27 vs. +16%, *p* = 0.5, ES = 0.2) (Figure 2; Table 2) and circulating levels of fibrinogen (−23 vs. −13%, *p* = 0.7, ES = −0.1), e-selectin (−6 vs. −5%, *p* = 0.7, ES = −0.03), and VCAM-1 (+8 vs. +20%, *p* = 0.3, ES = −0.3). ω-3 showed a tendency toward reduced ICAM-1 levels (+3 vs. +48%, *p* = 0.1, ES = −0.7) when compared with PL (Table 2).

**Table 2:** Endothelial function, inflammatory parameters, and lipid profile before and after the intervention in ω-3 and PL.

	PL (*n* = 11)			ω-3 (*n* = 11)			PL vs. ω-3		
	Pre	Post	Δ (95% CI)	Pre	Post	Δ (95% CI)	Δ difference (95% CI)	*p*	ES
**Inflammatory markers**									
C-reactive protein (mg/l)	3.6 ± 3.7	2.0 ± 1.0	−1.7 (−2.4 to −1.0)	4.4 ± 3.5	2.2 ± 1.8	−1.7 (−2.3 to −0.9)	−0.06 (−1.02 to 0.91)	0.9	−0.3
IL-6 (pg/ml)	0.84 ± 0.51	0.73 ± 0.46	−0.14 (−0.39 to 0.11)	1.32 ± 0.95	1.02 ± 0.61	−0.16 (−0.39 to 0.07)	0.02 (−0.32 to 0.36)	0.9	−0.4
IL-10 (pg/ml)	1.70 ± 0.57	2.17 ± 1.21	0.69 (0.40 to 0.99)	2.18 ± 1.74	2.10 ± 1.68	−0.07 (−0.35 to 0.20)	0.77 (0.37 to 1.17)	0.001	−0.9
TNF (pg/ml)	2.15 ± 1.00	2.16 ± 0.90	−0.01 (−0.19 to 0.17)	2.43 ± 0.84	2.10 ± 0.75	−0.30 (−0.49 to −0.10)	0.29 (0.01 to 0.56)	0.04	−0.95
IL-1-ra (pg/ml)	49.3 ± 64.3	20.6 ± 12.3	−22.3 (−44.8 to 0.2)	48.7 ± 55.9	32.8 ± 44.5	−11.6 (−34.1 to 10.9)	−10.7 (−42.5 to 21.1)	0.5	0.04
IL-1β (pg/ml)	0.90 ± 0.49	1.01 ± 0.60	0.08 (−0.22 to 0.38)	1.19 ± 0.68	0.92 ± 0.54	−0.18 (−0.49 to 0.11)	0.27 (−0.15 to 0.69)	0.2	−0.7

**Lipid profile**									
Triglycerides (mg/dl)	99.8 ± 27.8	81.5 ± 25.3	−23.0 (−37.8 to −8.2)	113.3 ± 62.2	91.0 ± 19.1	−16.5 (−30.3 to −2.7)	−6.5 (−26.7 to 13.7)	0.5	−0.06
LDL-chol (mg/dl)	104.4 ± 23.2	104.1 ± 26.9	−0.3 (−8.0 to 7.4)	107.7 ± 29.8	119.6 ± 21.9	12.4 (4.7 to 20.1)	−12.7 (−23.6 to −1.8)	0.02	0.85
HDL-chol (mg/dl)	45.9 ± 7.5	44.8 ± 7.9	−1.1 (−6.5 to 4.3)	46.0 ± 9.1	49.1 ± 13.2	3.1 (−2.3 to 8.5)	−4.2 (−11.9 to 3.5)	0.3	0.45
LDL/HDL ratio	2.35 ± 0.73	2.38 ± 0.71	0.0 (−0.3 to 0.3)	2.44 ± 0.83	2.62 ± 0.94	0.2 (−0.1 to 0.5)	−0.17 (−0.61 to 0.27)	0.4	0.32
Total cholesterol (mg/dl)	169.7 ± 24.5	165.8 ± 31.5	−4.3 (−16.8 to 8.3)	176.5 ± 34.8	186.2 ± 27.0	11.4 (−1.1 to 24.0)	−15.7 (−33.5 to 2.1)	0.07	0.67

**Markers of endothelial cell activation**									
Fibrinogen (mg/dl)	357 ± 71	310 ± 60	−98 (−155 to −43)	396 ± 134	307 ± 65	−85 (−145 to −24)	−14 (−96 to 69)	0.7	−0.11
e-selectin (ng/ml)	108 ± 46	103 ± 45	−1.0 (−23.7 to 21.6)	93 ± 39	88 ± 41	−6.6 (−28.3 to 15.0)	5.6 (−25.7 to 36.9)	0.7	−0.03
ICAM-1 (ng/ml)	635 ± 488	940 ± 608	274 (35 to 513)	712 ± 584	695 ± 457	29 (−199 to 256)	245 (−85 to 576)	0.1	−0.68
VCAM-1 (ng/ml)	657 ± 253	792 ± 339	112 (−41 to 265)	583 ± 276	631 ± 184	12 (−132 to 157)	99 (−111 to 309)	0.3	−0.25

**Safety**									
INR	2.0 ± 0.8	1.7 ± 0.7	−0.3 (−0.8 to 0.3)	2.3 ± 0.7	2.1 ± 0.7	−0.1 (−0.6 to 0.4)	−0.16 (−0.92 to 0.59)	0.7	−0.17

**Endothelial function estimates**									
RHI	2.08 ± 0.34	1.83 ± 0.53	−0.28 (−0.56 to −0.01)	1.80 ± 0.42	2.03 ± 0.36	0.08 (−0.18 to 0.34)	−0.37 (−0.7 to 0.01)	0.06	0.9
LnRHI	0.72 ± 0.16	0.56 ± 0.31	−0.19 (−0.34 to −0.05)	0.56 0.24	0.69 ± 0.17	0.05 (−0.09 to 0.18)	−0.24 (−0.44 to −0.04)	0.02	1.0
AI	−1.8 ± 14.1	−2.1 ± 13.7	−1.4 (−9.1 to 6.4)	7.7 ± 14.2	9.8 ± 20.0	3.4 (−4.1 to 11.6)	−4.7 (−16.0 to 6.5)	0.5	0.21

*Data expressed as mean ± SD. Delta change (Δ) and 95% confidence interval (95% CI), estimated difference between delta changes (Δ difference) and 95% CI, and level of significance (*p*) calculated using a mixed model adjusted by pre-values: effect size (ES)*.

*IL, interleukin; TNF, tumor necrosis factor; LDL, low-density lipoprotein; HDL, high-density lipoprotein; INR, international normalized ratio; ICAM-1, intercellular adhesion molecule-1; VCAM-1, vascular adhesion molecule-1; INR, international normalized ratio; RHI, reactive hyperemia index; LnRHI, reactive hyperemia index after natural log transformation; AI, augmentation index*.

### Inflammatory Profile

After the intervention, no significant differences between ω-3 and PL were observed in circulating levels of CRP (−51 vs. −45%, *p* = 0.9, ES = −0.3), IL-6 (−23 vs. −13%, *p* = 0.9, ES = −0.4), IL-1ra (−33 vs. −58%, *p* = 0.5, ES = 0.04), and IL-1β (−22 vs. +12%, *p* = 0.2, ES = −0.7). By contrast, ω-3 showed decreased circulating levels of IL-10 (−4 vs. +45%, *p* = 0.001, ES = −0.9) and TNF (−14 vs. +0.3%, *p* = 0.04, ES = −0.95) when compared with PL (Table 2).

### Lipid Profile

Following the intervention, ω-3, when compared with PL, showed increases in total cholesterol (+6 vs. −2%, *p* = 0.07, ES = 0.7) and LDL-cholesterol (+11 vs. −0.3%, *p* = 0.02, ES = 0.8). It is notable that before the intervention, only one patient in PL and three patients in ω-3 presented borderline levels of total cholesterol (>200 and <239 mg/dl), whereas one patient in PL and two patients in ω-3 presented borderline levels of LDL-cholesterol (>120 and <159 mg/dl). After the intervention, two patients in PL and four patients in ω-3 presented borderline levels of total cholesterol, whereas two patients in PL and four patients in ω-3 presented borderline levels of LDL-cholesterol. Importantly, these changes were not different within or between groups (*p* > 0.05). None of the patients presented high levels of total cholesterol (>240 mg/dl) or LDL-cholesterol (>159 mg/dl) (27) at any time. Moreover, no significant differences were observed between ω-3 and PL in HDL-cholesterol (+7 vs. −2%, *p* = 0.3, ES = 0.5), LDL-cholesterol/HDL-cholesterol ratio (+7 vs. +1%, *p* = 0.4, ES = 0.3), and triglycerides (−20 vs. −18%, *p* = 0.5, ES = −0.06).

### Dietary Intake

There were no significant differences for total energy, macronutrient, EPA, DHA, and ALA intake (without accounting for supplementation) between groups (*p* > 0.05 for all variables, Table 3).

**Table 3:** Dietary intake before and after the intervention in ω-3 and PL.

	PL (*n* = 11)			ω-3 (*n* = 11)			PL vs. ω-3		
	Pre	Post	Δ (95% CI)	Pre	Post	Δ (95% CI)	Δ difference (95% CI)	*p*	ES
Total energy (kcal)	1,567 ± 272	1,528 ± 665	−146 (−820 to 528)	1,738 ± 370	1,771 ± 406	62 (−476 to 599)	−208 (−1,071 to 654)	0.6	0.12
Protein (g)	61.1 ± 20.6	80.1 ± 33.5	0.1 (−31.9 to 32.0)	80.7 ± 23.8	77.1 ± 17.6	5.7 (−19.0 to 30.5)	−5.7 (−46.1 to 34.8)	0.8	−0.6
Protein (%)	15.2 ± 4.2	21.7 ± 7.6	−2.0 (−5.6 to 9.6)	19.1 ± 3.3	18.1 ± 4.6	1.2 (−5.0 to 7.4)	0.8 (−8.9 to 10.6)	0.8	−0.8
Carbohydrate (g)	223.0 ± 31.5	190.9 ± 94.1	−32.4 (−115.6 to 50.7)	219.5 ± 63.5	229.9 ± 62.4	6.8 (−63.3 to 77.0)	−39.2 (−148.1 to 69.5)	0.4	−0.5
Carbohydrate (%)	58.2 ± 7.0	48.2 ± 6.2	−4.2 (−11.8 to 3.4)	50.1 ± 7.5	51.6 ± 4.6	−1.4 (−7.4 to 4.5)	−2.8 (−12.4 to 6.9)	0.5	0.9
Fat (g)	47.8 ± 16.5	49.2 ± 19.4	−5.8 (−29.7 to 18.1)	59.7 ± 17.3	60.3 ± 16.6	6.2 (−13.1 to 25.6)	−12.1 (−42.9 to 18.7)	0.4	0.07
Fat (%)	26.6 ± 4.8	30.1 ± 4.5	−2.0 (−11.0 to 6.9)	35.7 ± 18.5	30.3 ± 4.5	−1.4 (−6.6 to 3.7)	−0.6 (−10.9 to 9.8)	0.9	−0.5
EPA (mg)^a^	8.0 ± 0.8	6.7 ± 5.0	−0.3 (−1.0 to 1.0)	9.5 ± 5.4	8.0 ± 6.7	−3.0 (−7.2 to 0.5)	3.0 (−6.9 to 13.1)	0.5	−0.4
DHA (mg)^a^	21.3 ± 22.0	11.3 ± 13.6	−12.6 (−26.5 to 1.1)	25.9 ± 21.9	13.7 ± 16.2	−13.2 (−24.8 to 1.6)	0.6 (−17.5 to 18.6)	0.9	−0.2
ALA (mg)	326 ± 476	290 ± 304	−74 (−299 to 151)	481 ± 263	473 ± 244	79 (−113 to 271)	−153 (−448 to 143)	0.3	0.2

*Data expressed as mean ± SD. Delta change (Δ) and 95% confidence interval (95% CI), estimated difference between delta changes (Δ difference) and 95% CI, and level of significance (*p*) calculated using a mixed model adjusted by pre-values: effect size (ES)*.

*EPA, eicosapentaenoic acid; DHA, docosahexanoic acid; ALA, α-linoleic acid*.

*^a^Values without accounting for supplementation*.

### Adverse Effects

There were no differences between ω-3 and PL in INR (−13.5 vs. −11.5%, *p* = 0.7, ES = 0.17) (Table 2). Importantly, all patients were within targeted INR before and after the intervention. There were no self-reported adverse events throughout the trial.

## Discussion

To the best of our knowledge, this is the first study to assess the safety and efficacy of n-3 PUFA supplementation in APS. Our main findings are that 16 weeks of n-3 PUFA supplementation was safe and improved endothelial function of patients with well-controlled PAPS.

Endothelial dysfunction mediated by aPLs has been associa-ted with an increased risk of thrombosis, accelerated atherosclerosis, myocardial infarction, and stroke in patients with APS (5). Thus, strategies capable of minimizing this burden may be valuable in the management of this disease.

In this study, we showed that 16 weeks of n-3 PUFA supplementation (3 g of EPA and DHA/day) led to improvements in endothelial function estimates in PAPS patients. Although mean improvements may be considered modest, half of the patients in ω-3 showed improvements in endothelial function estimates above the previously reported CVs (>30% change in RHI), whereas half of the patients in PL showed decreases in endothelial function estimates above the previously reported CVs (>25% change in RHI) (Figure 2). Importantly, this was observed in patients with similar physical activity levels and no changes in food intake, including n-3 PUFA intake, throughout the intervention, which are important confounding factors. It is worth noting that increases in RHI similar to those observed in the present study have been reported in response to pharmacological (anti-inflammatory drugs) (28) and nonpharmacological treatments (antioxidant-rich foods and physical exercise) (29–32) in previous studies. Thus, the beneficial effects of n-3 PUFA supplementation on endothelial function in our APS patients may be considered clinically relevant.

Our results corroborate the beneficial effects of marine-derived n-3 PUFA supplementation on endothelial function in patients with conditions associated with accelerated atherosclerosis, such as T2D, dyslipidemia, and obesity (11, 12, 14, 33). The exact mechanisms underlying this effect remain elusive. Nonetheless, the incorporation of n-3 PUFAS into cell membrane phospholipids seems to play a fundamental role as it modulates a number of cellular functions, including signal transduction, protein and membrane trafficking, and ion channel kinetics, which could beneficially impact endothelial function (34).

In this regard, marine n-3 PUFA supplementation has been shown to increase endothelial nitric oxide synthesis (eNOS) *via* increased translocation of eNOS from cell membrane caveolin to the cytosol, leading to eNOS system activation and vasodilation (35, 36). Another possibility is that the anti-inflammatory effect of marine n-3 PUFA could positively affect endothelial function (37). This may occur *via* a lower incorporation of n-6 PUFA on cell membranes (due to a higher n-3 PUFA incorporation) (38, 39), ensuing a lower production of pro-inflammatory eicosanoids (e.g., two-series prostaglandins and four-series leukotrienes) (40). Moreover, EPA and DHA are precursors for not only less potent pro-inflammatory eicosanoids but also to anti-inflammatory and inflammation-resolving lipid mediators, namely resolvins, protectins, and maresins (the latter two being derived from DHA only) (41). These lipid mediators have been shown to reduce the infiltration of neutrophils to inflamed sites, activate macrophage phagocytosis of apoptotic cells, and reduce the production of the classic pro-inflammatory cytokines TNF and IL-1β (42–44). Finally, n-3 PUFA may also exert its anti-inflammatory effect *via* binding to the G-protein coupled cell membrane receptor 120 (GPR120) on macrophages, which inhibits the activation of nuclear factor kappa B (NF_κ_B), a well-known transcription factor involved in the upregulation of genes encoding pro-inflammatory cytokines (45). These anti-inflammatory effects of n-3 PUFA supplementation are thought to lead to a lower production of endothelial adhesion molecules and leukocyte–endothelial interaction (46), which are critical to the initiation of vascular inflammation, thus positively affecting endothelial function.

We observed a tendency toward a reduced ICAM-1 response to n-3 PUFA supplementation when compared with placebo controls. This is in concert with a meta-analysis showing that n-3 PUFA supplementation reduces ICAM-1, but not VCAM-1 or e-selectin, in healthy and dyslipidemic individuals (47). VCAM-1 and e-selectin are expressed in endothelial cells upon cytokine activation, whereas ICAM-1 is expressed also in a number of immune cells (monocytes, macrophages, and lymphocytes). Based on this, the authors have suggested that n-3 PUFA selectively suppresses monocytes, rather than endothelial cells, leading to lower circulating levels of ICAM-1, but not the other markers of endothelial activation, which is corroborated by our data.

The selective suppression of monocytes, which leads to the downregulation of inflammatory cytokine secretion from these cells, may also explain the well-known anti-inflammatory effects of n-3 PUFA supplementation (48). Supplementation of ≥3 g/day of EPA and DHA has been shown to lead to reductions in TNF-circulating levels in T2D (49) and decreased TNF and IL-1β production by endotoxin-stimulated mononuclear cells of healthy individuals (50). This is in line with our current results of decreased levels of TNF in response to n-3 PUFA supplementation when compared with placebo. Notably, we also observed significant reductions in IL-10 levels in response to n-3 PUFA supplementation. Because IL-10 is a classic anti-inflammatory cytokine (51), this could be interpreted as detrimental. However, it is likely that the modest decrease in the pro-inflammatory cytokine TNF may have led to a downregulation of IL-10 secretion in a homeostatic manner, which may have contributed to the improvements in endothelial function in response to n-3 PUFA supplementation when compared with placebo.

Similar to previous findings (52), we observed modestly increased levels of total cholesterol and LDL-cholesterol in ω-3 after the intervention when compared with those in PL. However, the proportion of patients moving from desirable to borderline levels of total cholesterol and LDL-cholesterol was the same between groups. Moreover, the nonsignificant increase in HDL in response to n-3 PUFA supplementation led to an unchanged LDL-cholesterol/HDL-cholesterol ratio, a superior predictor of CVD risk than total cholesterol and LDL-cholesterol levels (53), which argues against a harmful effect. Finally, previous studies have demonstrated that n-3 PUFA-induced increase in LDL-cholesterol derives from an increase in large buoyant non-atherogenic LDL particles rather than that in small dense atherogenic LDL particles (54) due to a reduction in VLDL-cholesterol production which enhances the rate of conversion of VLDL-cholesterol to LDL-cholesterol particles (52).

Another safety concern was that n-3 PUFA supplementation could impair prothrombin time in APS patients. However, we did not observe any changes in INR in ω-3 in response to supplementation, and all patients remained within their targeted INR throughout the study, which suggests that n-3 PUFA supplementation in these patients does not seem to predispose to hemorrhagic episodes. Moreover, there were no self-reported adverse effects throughout the intervention. Altogether, these results attest to the safety of up to 4 months n-3 PUFA supplementation (3 g/day) in patients with well-controlled PAPS.

This study was not without limitations. This was a relatively small-scale study, likely with limited power to detect modest albeit potentially clinically relevant changes in secondary outcomes. Moreover, we cannot generalize our findings to APS patients with different disease severity, drug regimens, and comorbidities. In fact, our patients had well-controlled disease, as none of them had presented with hemorrhagic or thrombotic episodes in the year before entering nor during the study, and none of the patients had T2D, a condition associated with endothelial dysfunction and accelerated atherosclerosis (15). This rigid internal control to eliminate potential confoundable conditions, which could introduce interpretation bias, may partially explain the modest improvements in endothelial function observed in this study, as a ceiling effect may have occurred. In this regard, we cannot rule out the possibility that patients with a more severe disease or associated metabolic diseases might respond differently to the supplementation of n-3 PUFA, with greater improvements in systemic inflammatory markers and in endothelial function. Finally, this was a short-term study whose primary end point is a surrogate marker for endothelial function and, thus, CVD risk. Long-term follow-up studies with higher sample sizes and statistical power are warranted in order to evaluate if the observed changes in endothelial function will effectively lead to significant improvements in CVD morbidity and mortality and clinical features of the disease and to attest the safety of n-3 PUFA in APS.

In conclusion, our findings suggest that 16 weeks of n-3 PUFA supplementation was safe and led to improvements in endothelial function in patients with well-controlled PAPS. These results support the role of n-3 PUFA supplementation as an adjuvant therapy in APS focused on reducing an important cardiovascular risk factor.

## Ethics Statement

Clinical trial registered at http://ClinicalTrials.gov as NCT01956188. This manuscript is reported according to the CONSORT guidelines.

## Author Contributions

SF and FB conceived the study, analyzed and interpreted the data, and drafted the manuscript. SF, LS, MS, AH, BG, EB, AS-P, DA, KK, and MI acquired data. HR analyzed the data. All authors revised and approved the final version of the manuscript and are, thus, accountable for its content.

## Conflict of Interest Statement

The authors declare that the research was conducted in the absence of any commercial or financial relationships that could be construed as a potential conflict of interest.
